# Maximizing Production of Human Interferon-γ in HCDC of Recombinant *E. coli*

**Published:** 2013

**Authors:** Valiollah Babaeipour, Seyed Abbas Shojaosadati, Nader Maghsoudi

**Affiliations:** a *Biological Science and Technology Department, Malek Ashtar University, Tehran, Iran.*; b *Biotechnology Part, Department of Chemical Engineering, Faculty of Engineering,TarbiatModares University, Tehran, Iran.*; c *Neuroscience Research Center, Shaheed Beheshti University of Medical Sciences, Tehran, Iran.*; d *Department of Bioscience Engineering, Faculty of New Sciences and Technologies, Tehran University, Tehran, Iran.*

**Keywords:** Human interferon-*γ*, Fed-Batch, *Escherichia coli*, Specific growth rate, Induction condition

## Abstract

Tuning recombinant protein expression is an approach which can be successfully employed for increasing the yield of recombinant protein production in high cell density cultures. On the other hand, most of the previous results reported the optimization induction conditions during batch and continuous culture of recombinant *E. coli, *and consequently fed-batch culture have received less attention. Hence, in this research induction conditions for the over-production of recombinant interferon-*γ *including the amount of inducer, induction time and post-induction duration during chemical induction were optimized. *E. coli *BL21 (DE3) (pET3a-*hifnγ*) was used to over-express human interferon-gamma (hIFN-*γ*) in an exponential fed-batch procedure with a maximum attainable specific growth rate of 0.55 h^-1^ at the beginning of feeding and 0.4 h^-1^ in induction time. The factors were considered as the amount of inducer (IPTG) in the range of 0.565-22 mg g^-1^ L^-1^ at seven levels, cell density at induction time as 53, 65 and 75 g (dry cell weight) L^-1^, induction duration at different intervals of 3, 4, and 5 h after induction time.

The final concentration of biomass and interferon gamma reached to 127 g L^-1^ (DCW) and 51 g (hIFN-*γ*) L^-1^ after 17 h, and also the final specific yield and overall productivity were obtained 0.4 g (hIFN-*γ*) g^-1^ DCW and 3 g (hIFN-*γ*) L^-1^ h^-1^, respectively, which are the highest amounts of reported specific yield and productivity for recombinant proteins production.

## Introduction

HIFN-*γ *is a glycosylated protein with a total molecular size of 25 kDa which is composed of 143 amino acid residues (1-2). However, recombinant hIFN-*γ *(rhIFN-*γ*) expressed in *E.* *coli *is not glycosylated and has a molecular weight of 17 kDa, but is still active physiologically (3). *E. coli *is one of the most widely used hosts for production of heterologous proteins (4). Because most proteins are accumulated intracellularly in recombinant *E. coli *as inclusion bodies, productivity (and the specific productivity) is proportional to the final cell density (*i.e*. the production of product per amount of biomass per time). The high cell density cultivation (HCDC) techniques for cultivation of *E. coli *have been developed to improve productivity. Fed-batch processes have most often been used to obtain high cell densities (4-8).

Overall productivity of a recombinant product depends on both the biomass concentration and the specific cellular product yield. Whereas fed-batch processes primarily focus on increasing the biomass concentration, the culture conditions during growth and production phases may affect the specific cellular product yield. As culture conditions are related to the regulation of cloned gene expression, depending on host-vector system, it seems that they have a significant influence on the product yield of fed-batch processes employing recombinant microorganisms (4, 6, 9). Induction conditions can significantly affect the specific cellular product yield (6, 9-12). The amount of inducer, the strategy of its addition to the medium, culture conditions at the time of induction, induction time and post-induction duration have been reported to have important effects on the efficiency of induction (9-16). The amount of inducer required to titrate the repressor molecules is proportional to the total cell mass as well as the optimal specific concentration of the inducer, therefore it needs to be optimized for maximizing the recombinant protein synthesis at any cell concentration and any host (9-11, 13). The induction time is another important variable because maximum yield of foreign protein in fermentation depends on the point in the growth cycle at which expression is induced (14-16). For strains whose growth and/or viability is drastically reduced following induction, induction in late-log or stationary phase provides high cell densities for increased product formation (9, 14). 

However, the optimum induction strategy is generally determined through trial-and-error methods or by taking the effects of various culture conditions on the recombinant gene expression into account (9-16). Most of the previous results reported the optimization induction conditions during batch and continuous culture of recombinant *E. coli *and fed-batch culture have received less attention. It may not be often correlated to high cell density fed-batch culture because of considerable change of metabolic and physiological conditions of recombinant cells with increasing cell density (5, 6, 8, 17, 18). 

Also in our previous works, we developed a simple fed-batch process using a modified variable specific growth rate feeding strategy for high cell density cultivation of *E. coli *BL21 (DE3) expressing human interferon-gamma (hIFN-*γ*). In that strategy that the feeding rate was adjusted to achieve the maximum attainable specific growth rate during fed-batch cultivation, the final specific yield and overall productivity of recombinant were enhanced remarkably. Therefore, the present work focused on production rhIFN-*γ *more enhanced by tuning recombinant protein expression in fed-batch culture of *E. coli *which was developed in our earlier researches (22). Fed-batch culture has been performed by employing an exponential feeding in which value of specific growth rate was kept on maximum attainable level. We have reported here the super-production of rhIFN-*γ *by optimization of the amount of inducer, induction time and duration of production phase.

## Experimental

*Microorganism and vector system*

*E. coli *strain BL21 (DE3) (Novagen, UK) was used as host for rhIFN-*γ *expression. This strain was transformed with a commonly available plasmid pET3a, an inducible expression vector (Novagen), in which the hIFN-*γ *gene (Noor research and Education Institute, Tehran, I.R. Iran) was inserted into the *Not*I and *Nde*I sites. Host cells were transformed with the plasmid using the calcium chloride procedure (19). The transformed cells were spread on several LB agar plates containing 100 mg L^-1^ ampicillin.

*Media and solutions*

LB (Luria–Bertani) agar medium and M9 medium were used for plate cultivation and seed culture preparation *E. coli *strain BL21 (DE3). M9 modified medium contained (g l^-1^): glucose, 10; K_2_HPO_4_, 15; KH_2_PO_4_, 7.5; Citric acid, 2; (NH_4_)_2_SO_4_, 2.5; MgSO_4_.7H_2_O, 2; and trace element solution, 1 mL l^-1^ was used for fedbatch cultivation. Trace element solution had a composition of (g L^-1^ in 1M HCl): FeSO_4_.7H_2_O, 2.8; MnCl_2_.4H_2_O, 2; CoSO_4_.7H_2_O, 2.8; CaCl_2_.2H_2_O, 1.5; CuCl_2_.2H_2_O, 0.2; ZnSO_4_.7H_2_O, 0.3 g (19).

 Feed medium in fed-batch experiments contained (g L^-1^): glucose, 700; MgSO_4_.7H_2_O, 20; and trace element solution, 5 mL L^-1^. 

The fed-batch cultivation was carried out in a 2 L bench-top stirred bioreactor (Inforse AG Ch- 4103, Switzerland) with working volume of 1 l and including two six-blade Rushton impellers with speed range of 50-1200 rpm.

*Analytical methods*

Cell growth was monitored by measuring culture optical density (OD) at 600 nm and dry cell weight (DCW). In order to determine cell dry weight, 5 mL of fermented broth was centrifuged at 9000 rpm for 10 min, washed twice with 9% (w/v) NaCl isotonic solutions and dried at 105°C until constant weight. 

Glucose, ammonia, phosphate and acetate were analyzed enzymatically by using various kits (ChemEnzyme CO., I.R. Iran; Boehringer Mannheim/R-Biopharm, Germany).

The expression level of rhIFN-*γ *was determined by SDS-PAGE using 12.5 % (w/v) poly-acrylamid gels. Gels were stained with Coomassie brilliant blue R250 and then quantified by gel densitometer. Total soluble protein was analyzed by Bradford method with Bovine Serum Albumin as standard (20) and rhIFN-*γ *was measured by ELISA assay (21). 

The stability of the plasmid in the recombinant *E. coli *strain was determined by aseptically sampling from the bioreactor at different cell densities. Fermentation broth samples, diluted with 9% (w/v) NaCl when required were plated on LB agar plates with and without ampicillin supplementation (three replicates for each case). The fraction of plasmid-containing cells was calculated as the average ratio of viable colonies on LB with ampicillin to those on LB without antibiotic (22).

*Cultivation procedure*

The initial batch culture was started by the addition of 100 mL of overnight incubated seed culture (OD_600_ = 0.8 ± 0.1) into bioreactor containing 900 mL of M9 modified medium. The pH was maintained at 7 by the addition of 25 % (w/v) NH_4_OH or 3M H_3_PO_4_ solutions. Dissolved oxygen was controlled at 30-40% of air saturation by the control of both agitation rate and the inlet air, which was enriched with pure oxygen if necessary. Foam was controlled by the addition of silicon-antifoaming reagent. After the depletion of initial glucose in the medium, as indicated by the rapid increase in the dissolved oxygen concentration, the feeding was initiated. The Feeding rate was increased stepwise based on exponential feeding strategy with maximum attainable specific growth rate during fed-batch cultivation. The exponential feeding was determined by using Equation 1 (23).

M (t) = F (t) S0 = (μ (t) / Y _x/s_ + m) V_0_ X_0_ exp.

$\int_{\mathrm{t0}}^{t}\mu (t)\mathrm{dt})$                    (Equation 1)

Here, V_0_ is the medium volume in the bioreactor (l), X_0_ g (DCW) l^-1^ is the biomass concentration at the start of feeding, t is the time (h), μ is the specific growth rate (h^-1^), S_0_ is the glucose concentration (g L^-1^) in the feeding solution, F(t) is the feeding rate (h^-1^), M(t) is the mass feeding rate (g h^-1^), Y _x/s_ (g DCW g^-1^ glucose) is the yield of biomass on substrate (glucose), t_0_ (h) is the starting time for each feeding step, m is the specific maintenance coefficient (g g^-1 ^h^-1^). The yield coefficient (Y_ x/s_) and maintenance coefficient (m) were set at 0.5 and 0.025 g g^-1^ h^-1^ (22), respectively.

## Result and Discussion

*Experimental design*

The factors were considered as the amount of inducer (IPTG) in the range of 0.565-22 mg g^-1^ L^-1^ at seven levels, each level being two times in proportion to previous one, and cell density at induction time as 53, 65 and 75 g (dry cell weight) L^-1^. At each set of experiments, samples of 2-5 h after induction were hourly analyzed for rhIFN-*γ *production to find the effect of postinduction duration. First, the amount of inducer was optimized during fed-batch processes while recombinant bacterial cells induced at dry cell weight of 65 g L^-1^. Then the optimum time of induction was obtained by performing induction at two levels adjacent to the optimum value of the inducer concentration. The optimum induction duration was obtained at different intervals of post-induction.

*Effect of inducer concentration on cell growth and rhIFN-γ production*

In previous experiments in batch and fedbatch cultures, the concentration of IPTG 3 mmole L^-1^ was used for a complete titration of repressor molecules (19, 21, 22). Although the IPTG quantity used for expression in Lacbased promoters systems are often given in mmol L^-1^, for high cell density cultures, it is regarded more appropriate to express the IPTG quantity for induction by gram of dry biomass rather than in volumetric units. The calculated value from previous batch and fed-batch culture experiments was 3 mmol L^-1^ ≡ 11 mg g^-1^ L^-1^ (IPTG per DCW) and was used as a reference parameter for the initial value of fed-batch induction experiments. Remarkably, it was possible to obtain the significant level of cell growth and recombinant protein production in fed-batch and batch mode at the same IPTG concentration, but cell growth was ceased 2-3 h after induction and consequently it was not possible to obtain the maximum attainable cell growth and protein production (21, 22).

**Figure 1:**
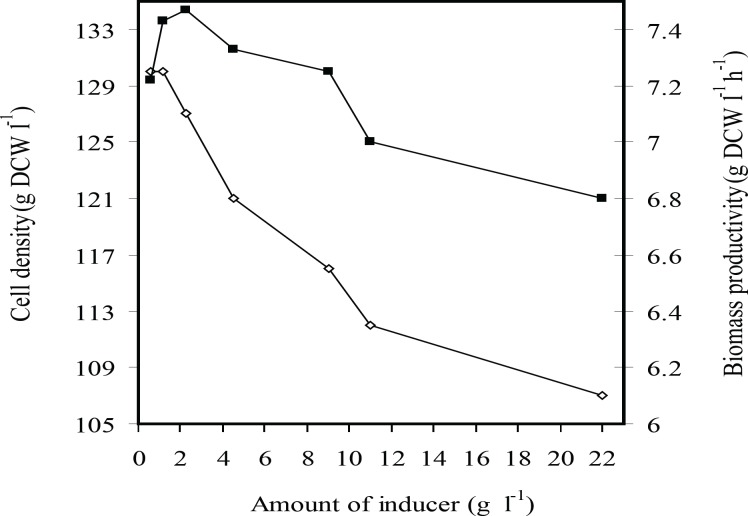
The effects of inducer concentration (g L^-1^ g^-1^ IPTG per DCW) on the final cell density (g L^-1^ DCW) (■) and biomass productivity (gL^-1^ h^-1^ DCW) (□) at fed-batch cultures of *E.coli* BL21 (DE3) (pET3a-*ifnγ*).

**Figure 2:**
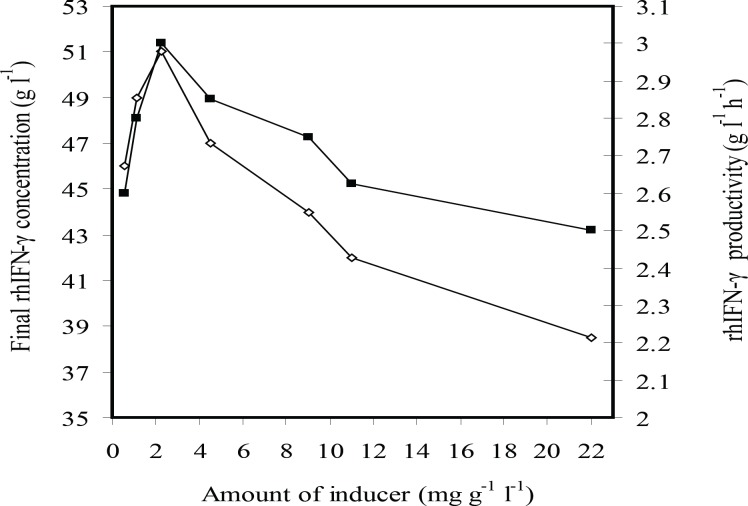
The effects of inducer concentration (g L^-1^ g^-1^ IPTG per DCW) on the rhIFN-*γ *production (g L^-1^ rhIFN-*γ*) (■) and rhIFN-*γ *productivity (g L^-1^ h^-1^ rhIFN-*γ*) at fed-batch cultures of *E.coli *BL21 (DE3) (pET3a-*ifnγ*)*.*

Figures 1 and 2 show the effects of inducer concentration on cell growth and rhIFN-*γ* production in fed-batch culture of *E. coli *BL21 (DE3) (pET3a-*hifnγ*) which induced at dry cell weight of 65 g L^-1^. It was seen that the final cell density and biomass productivity increased by decreasing inducer concentration from 22 to 2.25 mg g^-1^L^-1^. By reduction (the) amount of inducer to 0.565 mg g^-1^L^-1^, the final cell density remained constant while biomass productivity declined. 

Figure 2 indicates that the final concentration of rhIFN-*γ *and rhIFN-*γ *productivity were enhanced by decreasing (the) amount of inducer from 22 to 2.25 mg g^-1^L^-1^, but then diminished by the deduction of the inducer concentration to 0.565 mg g^-1^L^-1^.

**Figure 3:**
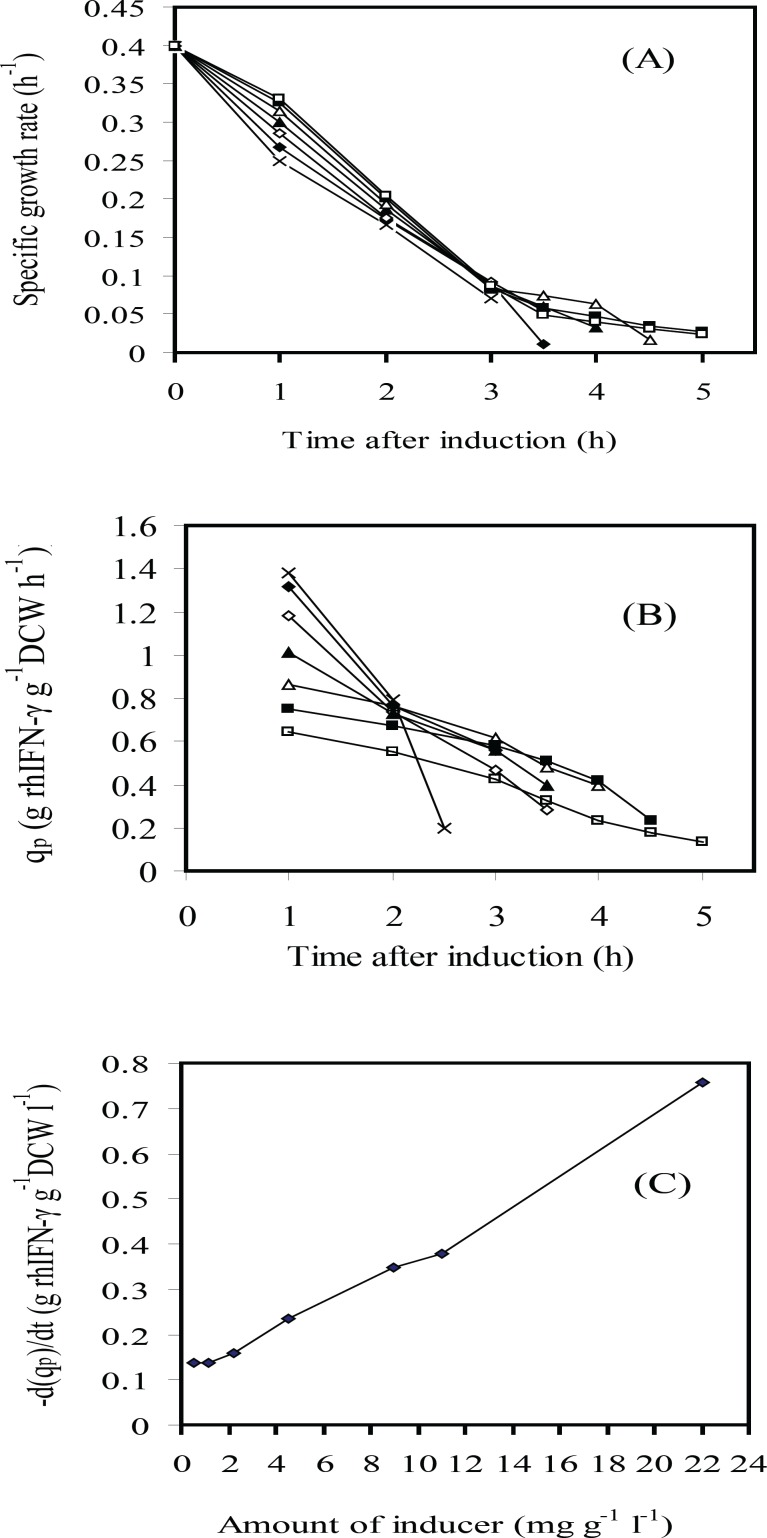
Time courses of several variables after induction: (A) specific growth rate (μ); (B) rhIFN-*γ *production rate (*q*_p_) and (C) rhIFN-*γ *production rate variation d(*q*_p_)/d*t *vs. IPTG concentration (g L^-1^ g^-1^ IPTG per DCW): 22(×); 11(♦); 9(◊); 4.5 (▲); 2.25(Δ); 1.13(■); 0.565(□)at fed-batch cultures of *E.coli* BL21 (DE3) (pET3a-*ifnγ*).

**Figure 4:**
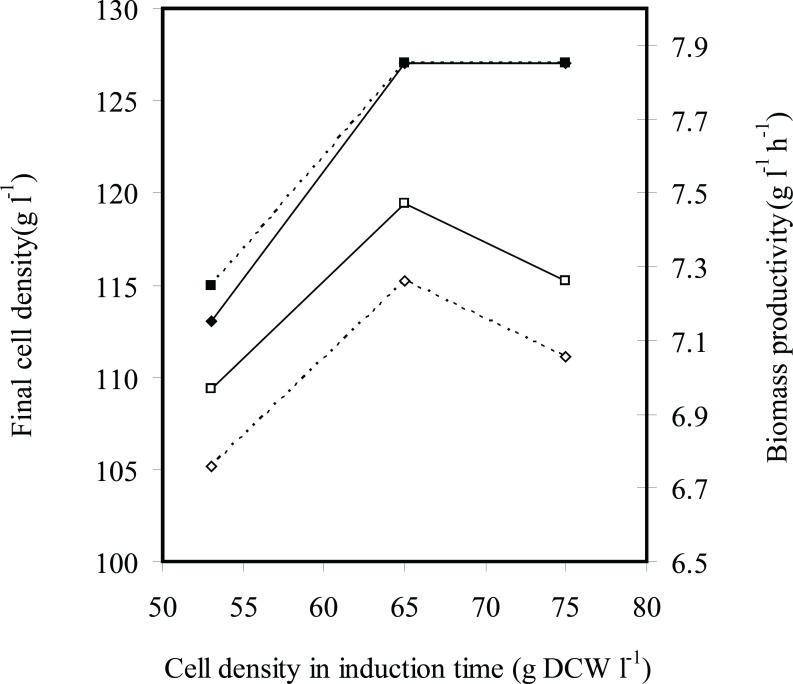
The effects of induction time (cell density at induction time g L^-1^ DCW) on the final cell density (g L^-1^ DCW) (■) and biomass productivity (g L^-1^ h^-1^ DCW) (□) in fed-batch cultures of *E.coli *BL21 (DE3) (pET3a-*ifnγ*). The black and dotted lines denote inducer concentrations of 2.25×10^-3^ and 1.13×10^-3^ g L^-1^ g^-1^ IPTG per DCW, respectively

For understanding the phenomenon concerning the cell growth, rhIFN-*γ *production level and IPTG concentration, the profiles of specific growth rate and specific rhIFN-*γ* production rate (*q*_p_) were depicted in Figures 3 and 4. The results indicated that the postinduction specific growth rate decreased for all IPTG assayed especially at higher IPTG concentrations at the first two hours after induction (Figure 3A). The *q*_p_ also decreased progressively after induction (Figure 3B). The decay rate (-d(*q*_p_)/d*t*) was directly proportional to the volumetric IPTG concentration essayed (Figure 3C). In summary, growth and protein production rates decreased with the increase of IPTG concentration. The results showed that there were not a direct relation between concentration of inducer and expression of the recombinant protein. Indeed, the overall level of rhIFN-*γ *expression in fed-batch culture involved a trade-off between cell yields and specific rhIFN-*γ *production rate. As a result, use of an intermediate level of inducer *i.e*. 4.5-1.13 mg g^-1^ L^-1^ (IPTG per DCW) was necessary in order to maximize the overall expression of the recombinant protein. Therefore, the optimal inducer concentration 2.25 mg g-1 L-1 (IPTG per DCW) was chosen.

*Effect of induction time on cell growth and rhIFN-γ production*

Both determination of optimal induction time and inducer concentration are important factors for increasing the overall productivity of recombinant protein in HCDC of recombinant *E.* *coli *(5). While the applied level of IPTG inducer can be varied to adjust the extent of the metabolic burden imposed on the cell, the maximum yield of foreign protein from fermentation will also depend on the point in the growth cycle at which expression is induced (9). 

The effect of induction time on cell growth and rhIFN-*γ *production was studied after optimization of IPTG concentration. For this reason, induction was carried out at three different intervals with cell densities of 53, 65 and 75 gL^-1^ at optimized IPTG concentration 2.25 mg g^-1^L^-1^ as well as adjacent point of 1.13 mg g^-1^ L^-1^ (IPTG per DCW). The induction time was selected according to the growth curve of fed-batch culture *E. coli *BL21 (DE3) (pET3a*hifnγ*) for non-induction condition (22). The specific growth rate at cell densities 53-65 g L^-1^ slightly varies of 0.42-0.4 h^-1^, while it decreases to 0.35 h^-1^ at cell densities 65-75 gL^-1^. On the other hand, recombinant *E. coli *is entered to decrease log phase at cell density of 65 g L^-1^.

**Figure 5:**
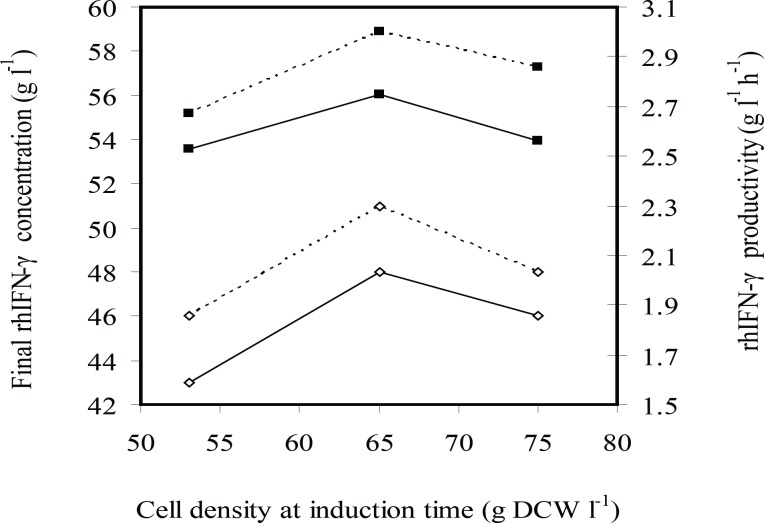
Effects induction time (cell density at induction time g L^-1^ DCW) on the rhIFN-γ production (g L^-1^ rhIFN-*γ*) (■) and rhIFN-*γ *productivity (g L^-1^ h^-1^ rhIFN-*γ*) at fed-batch cultures of *E.coli *BL21 (DE3) (pET3a-*ifnγ*). The black and dotted lines denote inducer concentrations of 2.25×10^-3^ and 1.13×10^-3^ g L^-1^ g^-1^ IPTG per DCW, respectively

Figures 4 and 5 indicate effects of induction time on cell growth and rhIFN-*γ *production, respectively. Final cell density was enhanced by increasing cell density of induction time of 53 to 65 g L^-1^ and (was) maintained constant. Maximum biomass productivity is achieved when induction was performed at cell density of 65 g L^-1^ with 2.25 mg g^-1^ L^-1^(IPTG per DCW). Maximum concentration and productivity of recombinant hIFN-*γ *were obtained with induction at cell density of 65 g L^-1^ and inducer concentration of 2.25 mg g^-1^ L^-1^(IPTG per DCW) (Figure 5).

At all experiments growth and/or viability of *E. coli *BL21 (DE3) (pET3a-*hifnγ*) was drastically decreased following the induction because of over-expression of recombinant protein. It seems that the induction at cell density of 53 g L^-1^ caused cell growth to stop by overexpression of rhIFN-*γ *before cell density reachesto maximum level and consequently final cell density, biomass productivity, concentration and productivity of rhIFN-*γ *decrease in comparison with the induction at 65 g L^-1^. 

On the other hand, reduction concentration and productivity of rhIFN-*γ *at cell density (of) 75 g L^-1^ in comparison with the induction at 65 g L^-1^ may be due to the bacterial cells that enter the stationary phase and consequently the protein production decreases. 

These results showed that optimal induction in the mid-log phase provided both high levels of rhIFN-*γ *and high density of cells to produce the maximum yield.

*Effect of post-induction duration on growth and rhIFN-γ production*

Although the post-induction duration highly affects concentration and overall productivity of recombinant protein in *E. coli*, it has received less attention in the literature (9, 25). 

Optimal post-induction duration in fed-batch culture of *E. coli *is affected by various factors, such as amount of inducer, induction time, strength of promoter, the response of the cell to recombinant protein expression, solubility of recombinant protein and characteristics of the protein itself as well as feeding strategy.

 In this study, post-induction duration of 4 h was selected according to previous results of batch and fed-batch culture of *E. coli *BL21 (DE3) (pET3a-*hifnγ*) (19, 21, 22) as base. Then, to obtain a proper post-induction duration at the all fed-batch experiments which were carried out for optimization inducer concentration and induction time, cell growth and protein production was followed at 1-5 h after induction. Finally, it was characterized that under optimal inducer concentration of 2.25 mg g^-1^ L^-1^ (IPTG per DCW) and induction time (DCW 65 g L^-1^), maximum productivity of rhIFN-*γ *can be obtained 4 h after induction.

*Kinetics of cell growth and rhIFN-γ production under optimum conditions*

**Figure 6:**
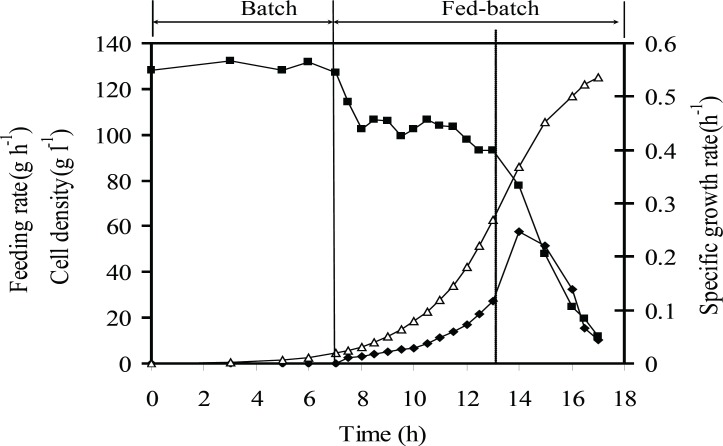
Growth kinetic of *E.coli *BL21 (DE3) (pET3a-*ifnγ*) under the optimum induction conditions. The dotted line indicates induction time. Specific growth rate (h^-1^) (■), cell density (g L^-1^ DCW) (Δ), feeding rate (g h^-1^) (♦). The dotted line indicates induction time

**Figure 7:**
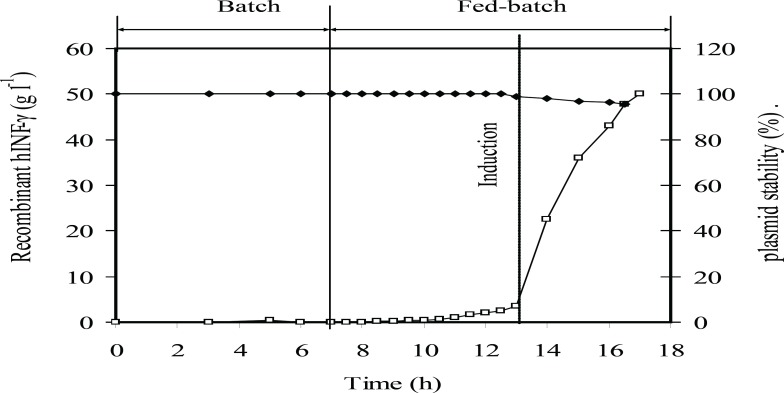
Kinetic of rhIFN-*γ *production at the optimum induction conditions at fed-batch culture of *E.coli *BL21 (DE3) (pET3a-*ifnγ*). Plasmid stability (%) (♦), and rhIFN-*γ *production (g L^-1^ rhIFN-*γ*) (□). The dotted line indicates induction time

Figures 6 and 7 show the kinetics of the recombinant *E. coli *growth and the rhIFN-*γ* production in the entire fed-batch process. By using exponential fed-batch procedure with a maximum attainable specific growth rate, under optimal induction conditions maximum cell density and rhIFN-*γ *concentration after 17 h were 127 g L^-1^ (DCW) and 51 g L^-1^ (rhIFN-*γ*), respectively. Also the maximum specific yield and productivity of rhIFN-*γ *were 400 mg (rhIFN-*γ*) g^-1^(DCW) and 3 g (rhIFN-*γ*) L^-1^h^-1^, respectively.

**Figure 8:**
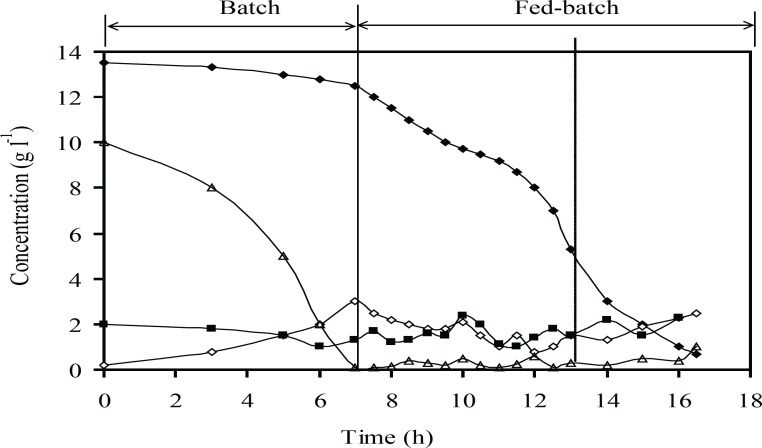
Concentrations of the main medium components (g L^-1^) include glucose (Δ), phosphate (♦), ammonium (■) and acetate (◊): at optimum induction conditions at fed-batch cultures of recombinant *E.coli *BL21 (DE3) (pET3a-*ifnγ*). The dotted line indicates induction time

Figure 7 showed that the plasmid stability was maintained at the highest level until the end of fed-batch process. A slight decrease in plasmid stability after induction is due to an increase in the metabolic burden of cells arising from recombinant gene expression. Figure 8 showed that concentrations of acetate, glucose, ammonium, and phosphate during fed-batch cultivation are lower than the inhibitory concentrations of these chemicals before and after the induction. 

As shown in Table 1 the results obtained in this study were compared with previous data of fed-batch culture of *E. coli *BL21 (DE3) (pET3a-*hifnγ*) under non-optimal induction condition (22). 

**Table 1:** Comparison results of fed-batch culture *E.coli *BL21 (DE3) (pET3a-*hifnγ*) under optimum condition (2.25 × 10^-3^ g g^-1^ l^-1^ IPTG per DCW, DCW = 65 g l^-1^ as induction time, post-induction duration 4 h) and non-optimized (11 mg g^-1^ l^-1^, DCW = 65 g l^-1^ as induction time, post-induction duration 3 h).

**Parameters**	**Optimum condition**	**Non-optimum condition** **(previous work) (22)**
Cultivation time (h)	17	16.5
Total glucose consumption (g)	295	312
Total ammonium solution 25%(w/v) consumed (g)	123	142
Final cell density (g l^-1^ DCW)	127	112
Productivity of biomass g l^-1^ h^-1^ (DCW)	7.65	7
Final concentration of rhIFN-γ (g l^-1^)	51	42
Yield of rhIFN-γ/ biomass, Y_p/x_ g g^-1^ (rhIFN-γ per DCW)	0.4	0.37
Yield of rhIFN-γ/ substrate, Y_p/s _g g^-1^ (rhIFN-γ per glucose)	0.17	0.16
Yield of biomass/ substrate, Y_x/s _g g^-1^ (DCW per glucose)	0.424	0.43
Productivity of rhIFN-γ g l^-1.^h^-1^	3	2.57

The comparison results showed the total final DCW increased from 112 g L^-1^ (22) to 127 g L^-1^. Also, the final concentration of rhIFN-*γ *increased from 42 to 51 g L^-1^, which shows about 20% increase with respect to the previous reported value. This led to a higher overall productivity of rhIFN-*γ *of 3 g L^-1^ h^-1^ compared to 2.58 g L^-1^ h^-1^. The high production of recombinant protein under optimum induction conditions is due to balancing the two opposing phenomena effective in maximizing the production of the recombinant protein (that is increasing the expression of target protein and decreasing cell growth) in such a way that the highest overall expression of the recombinant protein and the optimum cell density occurred at the same time. 

Also, the overall productivity of rhIFN-*γ* obtained in this study is by far higher than those reported by other researchers (5-8, 11, 12, 15, 19, 21-28). The increase in the level of overall productivity could be due to 1) recombinant protein production under induction optimum conditions 2) The reduction of process time, 3) increase in plasmid stability, 4) decrease in accumulation of by-products, especially acetate, 5) presence of nutrients (glucose, ammonium and phosphate) at a suitable concentration range during fed-batch cultivation, 6) higher ribosome content at higher growth rates (12, 28). 

In comparison with data reported by other researchers, the cultivation time in this process decreases while cell density, rhIFN-*γ *concentration, biomass and rhIFN-*γ* productivity increase significantly (4-8, 11- 15, 19, 21, 23-28). It may be due to the use of maximum attainable specific growth rate and maintaining it below the critical value during fed-batch cultivation which led to a desirable metabolic condition for cell growth and recombinant protein production. Plasmid stability is one of the most important issues affecting the productivity of recombinant protein production in *E. coli *fed-batch culture (17)*. *Although it seems that the variation of plasmid stability is a function of the specific growth rate, it has been difficult to find real trends in most cases. It has generally been observed that plasmid stability reduces with decreasing growth rate, primarily because the relative growth rate advantage of plasmid-free cells over those containing plasmids decreases under these conditions (17, 10). Hence, it can be expected that by increasing the specific growth rate, plasmid stability also increases (Figure 7).

 Acetate accumulation is a major challenge during production of recombinant protein at high cell density (21, 25, 29). Acetate formation can be minimized by controlling the specific growth rate below a certain value (depending on strain and medium composition) (4). The results presented in Figure 8 showed that the acetate concentration was lower than the reported inhibitory growth limit (less than 5 g L^-1^ for acetate). It could be due to the specific growth rate was controlled below the critical value and glucose concentration was maintained at a permissible range simultaneously, without any starvation and accumulation of glucose (Figure 8). The dissolved oxygen concentration was kept higher than minimum amount that was reported during fed-batch (more than 6% (v/v) air saturation) (30).

## Conclusion

This work demonstrates that i) induction conditions including induction time and duration, inducer concentration influenced significantly recombinant protein production in fed-batch culture *E. coli *ii) suitable induction conditions obtained in batch culture can not be correlated to fed-batch and Its necessary optimum conditions were obtained for any of fed-batch strategy and any of expression system iii) maximum productivity of recombinant proteins in fedbatch culture of *E. coli *is obtained when the opposing phenomena of increasing target protein expression and decreasing cell growth balanced by optimizing induction conditions, so that maximum expression of the recombinant protein and final cell density happened at the same time. Therefore, by adopting feeding strategy with maximum attainable specific growth rate (in the permissible range) during fed-batch culture of recombinant *E. coli *and optimization, induction conditions can be expected that maximum productivity of any recombinant protein achieved.
